# ADME profiling, molecular docking, DFT, and MEP analysis reveal cissamaline, cissamanine, and cissamdine from *Cissampelos capensis* L.f. as potential anti-Alzheimer's agents†

**DOI:** 10.1039/d4ra01070a

**Published:** 2024-03-25

**Authors:** Maram B. Alhawarri, Mohammad G. Al-Thiabat, Amit Dubey, Aisha Tufail, Dania Fouad, Bilal Harieth Alrimawi, Mohamad Dayoob

**Affiliations:** a Department of Pharmacy, Faculty of Pharmacy, Jadara University P.O.Box 733 Irbid 21110 Jordan m.hawarri@jadara.edu.jo; b School of Pharmaceutical Sciences, Universiti Sains Malaysia Gelugor 11800 Penang Malaysia Mohd.althiabat@gmail.com; c Department of Pharmacology, Saveetha Dental College and Hospital, Saveetha Institute of Medical and Technical Sciences Chennai-600077 Tamil Nadu India ameetbioinfo@gmail.com; d Computational Chemistry and Drug Discovery Division Quanta Calculus Greater Noida-201310 Uttar Pradesh India aishatufailansari@gmail.com; e Faculty of Dentistry, Ibn Sina University for Medical and Pharmaceutical Sciences Baghdad Iraq daniaalsafar2009@gmail.com; f Michael Sayegh Faculty of Pharmacy, Aqaba University of Technology Aqaba Jordan brimawi@aut.edu.jo; g Faculty of Pharmacy, MAHSA University Jenjarom Malaysia mohamaddayoob@mahsa.edu.my

## Abstract

The current pharmacotherapies for Alzheimer's disease (AD) demonstrate limited efficacy and are associated with various side effects, highlighting the need for novel therapeutic agents. Natural products, particularly from medicinal plants, have emerged as a significant source of potential neuroprotective compounds. In this context, *Cissampelos capensis* L.f., renowned for its medicinal properties, has recently yielded three new proaporphine alkaloids; cissamaline, cissamanine, and cissamdine. Despite their promising bioactive profiles, the biological targets of these alkaloids in the context of AD have remained unexplored. This study undertakes a comprehensive *in silico* examination of the binding affinity and molecular interactions of these alkaloids with human protein targets implicated in AD. The drug likeness and ADME analyses indicate favorable pharmacokinetic profiles for these compounds, suggesting their potential efficacy in targeting the central nervous system. Molecular docking studies indicate that cissamaline, cissamanine, and cissamdine interact with key AD-associated proteins. These interactions are comparable to, or in some aspects slightly less potent than, those observed with established AD drugs, highlighting their potential as novel therapeutic agents for Alzheimer's disease. Crucially, Density Functional Theory (DFT) calculations offer deep insights into the electronic and energetic characteristics of these alkaloids. These calculations reveal distinct electronic properties, with differences in total energy, binding energy, HOMO–LUMO gaps, dipole moments, and electrophilicity indices. Such variations suggest unique reactivity profiles and molecular stability, pertinent to their pharmacological potential. Moreover, Molecular Electrostatic Potential (MEP) analyses provide visual representations of the electrostatic characteristics of these alkaloids. The analyses highlight areas prone to electrophilic and nucleophilic attacks, indicating their potential for specific biochemical interactions. This combination of DFT and MEP results elucidates the intricate electronic, energetic, and electrostatic properties of these compounds, underpinning their promise as AD therapeutic agents. The *in silico* findings of this study shed light on the promising potential of cissamaline, cissamanine, and cissamdine as agents for AD treatment. However, further *in vitro* and *in vivo* studies are necessary to validate these theoretical predictions and to understand the precise mechanisms through which these alkaloids may exert their therapeutic effects.

## Abstract 2

Proaporphine alkaloids—cissamaline, cissamanine, and cissamdine—show promise against AD, with *in silico* studies highlighting their potential as new therapeutics.

## Graphical abstract

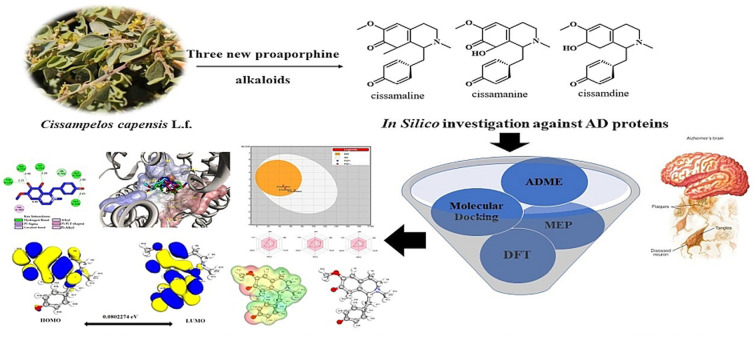

## Introduction

Alzheimer's disease (AD) is a prevalent neurodegenerative disorder that affects the brain, particularly the cortex and hippocampus, and is commonly observed in individuals over 65 years old.^1^ AD is characterized by a gradual decline in cognitive abilities, learning, and memory, leading to impaired bodily functions and eventual mortality.^2^ Projections of AD show an alarming increase of 82 million dementia cases by 2030 and 152 million by 2050.^2^ The pathophysiology of AD involves multiple changes, including acetylcholine (ACh) deficiency, amyloid plaque (Aβ) accumulation, phosphorylated tau proteins, and glutamatergic system imbalances.^1,3,4^ Recent studies have also emphasized the critical role of gamma-secretase (GS) in the amyloidogenic pathway, highlighting its importance in the cleavage of amyloid precursor protein (APP) and subsequent Aβ42 formation. This enzyme complex has become a target for therapeutic intervention, aiming to mitigate the Aβ accumulation central to AD pathology.^5,6^ The available five clinically approved drugs for AD, namely galantamine, memantine, tacrine, rivastigmine, and donepezil, target the modulation of the cholinergic system.^7^ Anticholinesterase drugs are primarily used to alleviate symptoms rather than cure the underlying disease in AD and other neurodegenerative disorders. However, these drugs can cause significant side effects, including gastrointestinal distress and fatigue, as well as dizziness, headaches, and muscle cramps.^1,2,7^

Naturally derived compounds have shown promise in treating neurodegenerative diseases like AD, due to their complex therapeutic properties and suitable pharmacokinetic profiles.^8^ Within this context, the *Cissampelos* genus of the Menispermaceae family, with 21 species native to various tropical and subtropical regions, has been traditionally employed for diverse medicinal purposes.^9^ Historically, various species of *Cissampelos* have been utilized in traditional medicine for the treatment of conditions such as asthma, arthritis, trauma and haemorrhage, and also for enhancing blood flow.^10^

**Fig. 1:**
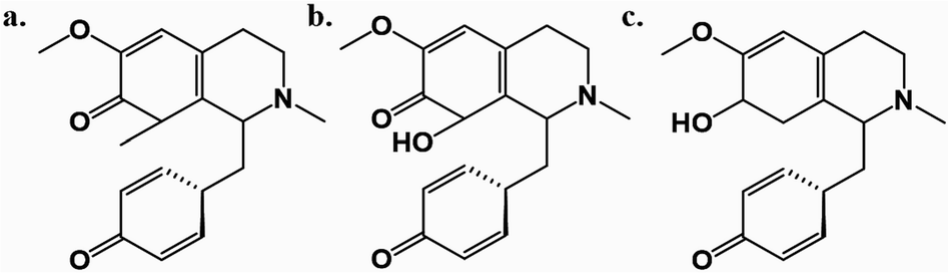
The chemical structures of cissamaline (a), cissamanine (b), and cissamdine (c).

Among these, *Cissampelos capensis* L.f. stands out in the Menispermaceae family for its widespread usage and has garnered interest due to its bioactive compounds and medicinal properties.^11^ This species is notably employed in the western regions of South Africa by the Khoisan and other rural communities, primarily as a remedy for blood purification and as a diuretic.^12^ Notably, a recent study highlighted the isolation of three new proaporphine alkaloids – cissamaline, cissamanine, and cissamdine – from the leaves of *C. capensis* (Fig. 1).^13^ Isoquinoline alkaloids, including proaporphine types, are recognized for their neuroprotective effects.^13^ However, the impact of these newly discovered proaporphine alkaloids on diseases like AD have not yet been studied experimentally or theoretically using molecular simulation techniques. This lack of information has led to the rationale of this study, which designed to fill this gap *via* utilizing computational strategies to investigate the putative neuroprotective properties of these compounds.

The aim of this study is to computationally investigate the potential of cissamaline, cissamanine, and cissamdine from *C. capensis* L.f. in combating AD. This includes evaluating their pharmacokinetic properties, binding affinities, and interactions with key Alzheimer's proteins. In addition, the study will investigate the electronic characteristics of these new alkaloids, offering insights into their reactivity and electronic structure. The results of this study may provide a valuable starting point for further research on the potential of new proaporphine alkaloids as treatments for AD and other related neurodegenerative conditions.

## Materials and methods

### ADME predictions

To assess the ADME properties of the proaporphine alkaloids—cissamaline, cissamanine, and cissamdine—we utilized the SwissADME web service, available at http://www.swissadme.ch (accessed on December 10, 2023). This computational tool enables the calculation of a broad spectrum of physicochemical descriptors and the estimation of ADME parameters, pharmacokinetic profiles, drug-likeness, and medicinal chemistry compatibility. We converted the two-dimensional chemical structures of the alkaloids into the SMILES (Simplified Molecular Input Line Entry System) format, which were then input into the web server for the predictive analysis.

### Molecular docking simulation

In this work, the aim was to investigate the potential of three new proaporphine alkaloids (cissamaline, cissamanine, and cissamdine) as natural hit molecules for AD using *in silico* approaches. These phytocompounds were utilized as ligands to evaluate their binding affinity against several human protein targets that have been suggested as therapeutically relevant in AD. These targets include angiotensin converting enzyme (ACE),^14^ β-site APP cleaving enzyme 1 (BACE1),^15^ glycogen synthase kinase-3 beta (GSK-3β),^16^ TNF-α converting enzyme (TACE),^17^ acetylcholinesterase (AChE),^18^ and gamma-secretase (GS)^6^ for AD. Modulation or inhibition of these protein targets may have the potential to impede or arrest the progression of neurodegenerative disorders, providing a promising avenue for further research.^19,20^

Crystal structures of five human protein targets (PDB IDs 1O86,^21^4DJU,^22^1Q5K,^23^3G42,^24^ and 4 M0E^25^) were initially downloaded from the RCSB Protein Data Bank (https://www.rcsb.org)^26^ on December 10, 2023. Subsequently, the crystal structure of the human GS complex with its inhibitor L-685458 (PDB ID: 7D8X)^27^ was added to our analysis and downloaded on March 1, 2024, to evaluate its interaction with the investigated compounds. Water molecules and heteroatoms were removed using Biovia Discovery Studio Visualizer.^28^ These proteins underwent preparation for molecular docking through the PDB2PQR web service (https://pdb2pqr.poissonboltzmann.org/pdb2pqr), accessed on December 12, 2023 and March 1, 2024, which included reconstructing missing atoms and assigning charges and radii based on the SWANSON force field (AMBER ff99 charges with optimized radii).^29,30^ Ionizable groups' protonation states were established using PROPKA3 at pH 7.40.^30,31^ Subsequently, protein structures were refined with MolProbity (http://molprobity.biochem.duke.edu/) on December 15, 2023 and March 1, 2024, for atom contact correction and hydrogen atom addition, enhancing structural analysis accuracy.^30,32^

The newly proaporphine alkaloids were sketched using PerkinElmer ChemDraw Professional 17.1 (PerkinElmer, Massachusetts, USA). Their structures subjected to energy minimization with the Molecular Mechanics 2 (MM2) force field in Chem3D 17.1 and were saved in PDB format for subsequent analyses. These alkaloids and the co-crystallized ligands from the protein targets were isolated and saved as PDB files using Biovia Discovery Studio Visualizer, with Gasteiger charges assigned *via* AutoDockTools 1.5.6.^33^ For validation, these co-crystallized ligands were re-docked to their respective proteins using AutoDock 4.2,^33^ providing a comparative baseline for the docking results of the new compounds.

AutoDock Tools 1.5.6 (The Scripps Research Institute, San Diego, CA, USA) facilitated the preparation of proteins and ligands for docking.^34^ Polar hydrogen and Kollman charges were added to the proteins, and Gasteiger charges to the ligands.^30^ These charged structures were saved in PDBQT format.^30^ Grid boxes were aligned with the active sites of the proteins (Table S1†). Docking simulations utilized AutoDock 4.2,^34^ with proteins set as rigid and ligands as flexible. We executed 100 docking runs, setting the population size at 150, the maximum evaluations at 2.5 million (medium), and the maximum generations at 27 000.^30^ The Lamarckian genetic algorithm guided these simulations,^35^ with most parameters remaining default and recorded in docking parameter files (DPFs). Molecular interactions between ligands and proteins were visualized and analyzed in both 2D and 3D using BIOVIA Discovery Studio Visualizer.

### Density function theory (DFT) and molecular electrostatic potential (MEP) calculations

Density Functional Theory (DFT) emerges as a dependable and cost-efficient technique for uncovering fundamental insights into material properties, ranging from energy profiles and geometric structures to electrical attributes and optical characteristics.^36^ This versatile approach proves essential for interpreting data across various scales, spanning from the microscopic realm of atoms and molecules to larger unit cells. In this study, DFT played a crucial role in thoroughly exploring parameters pivotal to electronic behavior, energetics, thermodynamics, and adsorption phenomena, with a specific emphasis on binding energy.^37,38^ Additionally, the investigation aimed to elucidate the reactivity of pharmacological complexes by employing quantum molecular descriptors, such as Highest Occupied Molecular Orbital (HOMO), Lowest Unoccupied Molecular Orbital (LUMO), band gap energy, chemical hardness, softness, electronegativity, and electrophilicity.^39–41^

The Gaussian 09 software package was utilized for the computational tool to optimize the geometry of molecular structures associated with proaporphine alkaloids—cissamdine, cissamaline, and cissamanine—employing the density functional three-parameter hybrid (B3LYP) methods along with the 6-311G (d, p) basis set.^42^ This meticulous approach ensured the derivation of precise and meaningful insights into the molecular characteristics, providing a profound understanding of the proaporphine alkaloids and their interactions within the pharmacological context. Furthermore, a Molecular Electrostatic Potential (MEP) diagram for the salt was generated using the B3LYP-D3/6-311G (d, p) method. This diagram serves as a visual representation of the Electrostatic Potential (ESP) superimposed onto the electron density (ED) surface, exhibiting a gradient of colors from the deepest red to the deepest blue. This graphical representation effectively communicates the electrostatic characteristics of the salt molecule, offering valuable insights into the distribution of electric charge across its surface.

## Results and discussion

### ADME properties of the new proaporphine alkaloids

**Fig. 2:**
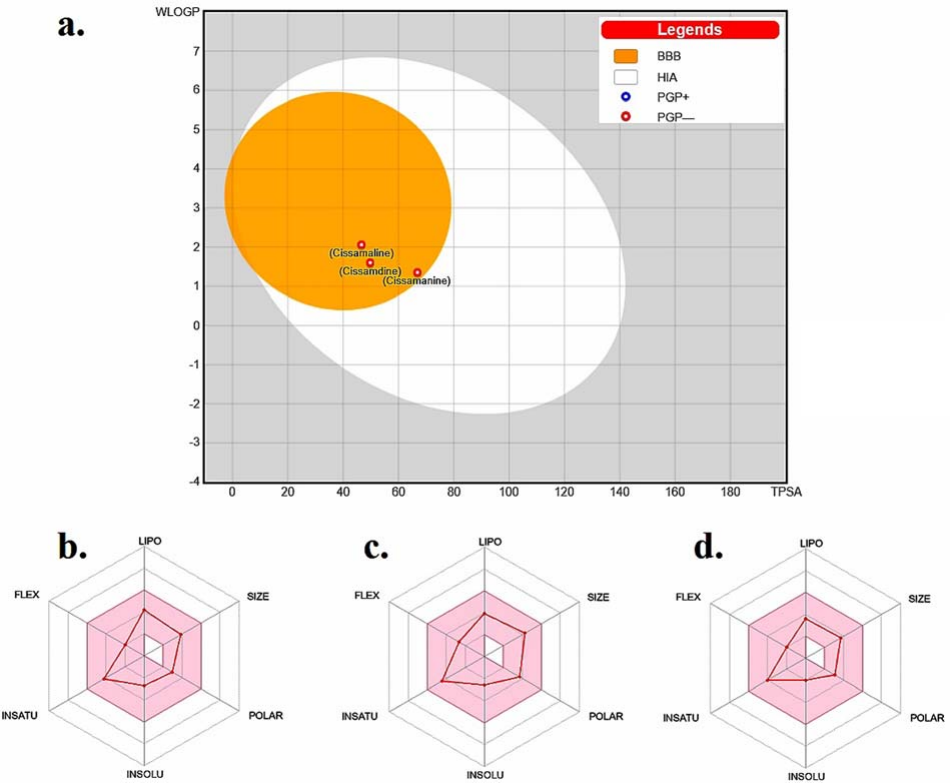
(a) Boiled-egg model depicting the blood–brain barrier permeability and oral bioavailability of the new proaporphine alkaloids: cissamaline, cissamanine, and cissamdine. The physicochemical property radars for (b) cissamaline, (c) cissamanine, and (d) cissamdine demonstrate their respective profiles, highlighting characteristics essential for CNS-active drug candidates. The colored zone is the suitable physiochemical space for oral bioavailability. Lipo (lipophilicity): −0.7 < WLOGP < +5.0. Size: 150 g mol^−1^ < MW < 500 g mol^−1^. POLAR (polarity): 20 Å^2^ < TPSA < 130 Å^2^. INSOLU (insolubility): −6 < log *S* (ESOL) < 0. INSATU (instauration): 0.25 < fraction Csp^3^ < 1. FLEX (flexibility): 0 < num. Rotatable bonds < 9.^43,44^

The “Boiled-Egg” model, presented in Fig. 2a, eloquently illustrates the potential of the new proaporphine alkaloids (cissamaline, cissamanine, and cissamdine) to penetrate the blood–brain barrier (BBB) and their oral bioavailability. The graphical representation situates cissamaline within the yolk region, indicating a high likelihood of BBB permeability and strong oral bioavailability, while cissamanine and cissamdine lie on the border, suggesting moderate attributes. The model predicates this prediction on the physicochemical properties of lipophilicity (LIPO) and the total polar surface area (TPSA), where a balance between hydrophobic and polar characteristics is crucial for brain uptake and oral absorption. The physicochemical radars in panels b, c, and d provide a nuanced view of each alkaloid, displaying a harmonious balance in their molecular size, lipophilicity, polarity, solubility, saturation, and flexibility. These characteristics are pivotal for a drug's pharmacokinetic profile, influencing absorption, distribution, metabolism, and excretion (ADME). Notably, the compounds fall within the acceptable range for molecular weight (<500 g mol^−1^) and rotatable bonds (<9), which are indicative of favorable drug-like properties.^45,46^

The penetration of the BBB is a formidable challenge in drug design, yet it is essential for therapeutic agents targeting CNS disorders. The balance between solubility and lipophilicity is a delicate one, as excessive lipophilicity can lead to poor solubility and *vice versa*. Moreover, the radar plots underline the compounds' structural flexibility, which is often associated with better interaction with biological targets. However, these predictions must be validated through *in vivo* pharmacokinetic and pharmacodynamic studies to ascertain their actual BBB penetration and bioavailability. The data presented suggests that cissamaline, cissamanine, and cissamdine could be promising candidates for further investigation, particularly in exploring their central effects and potential efficacy against AD. Their physicochemical profiles hint at an ability to reach central targets, which is a critical step in the development of new therapeutics for neurodegenerative.

Furthermore, an in-depth analysis of drug metabolism properties, with a focus on interactions with Cytochrome P450 enzymes,^47,48^ has been conducted and is presented in Table S2.† Our findings suggest that cissamaline, cissamanine, and cissamdine are not predicted to inhibit key enzymes such as CYP1A2, CYP2C19, CYP2C9, CYP2D6, and CYP3A4. The lack of inhibitory activity on these major CYP enzymes may imply a reduced potential for adverse drug–drug interactions mediated by CYP,^49^ a critical aspect in the development of AD therapeutics.^50^ These predictions of CYP enzyme interaction, along with the favorable BBB penetration characteristics, lend further support to the potential of these compounds as viable candidates for treating neurodegenerative diseases and underscore the need for additional empirical research.

### Molecular docking

Molecular docking aims to accurately model the structure of the ligand and the binding site, and to predict the molecular interaction binding pose of the ligand when it binds to the protein.^1^ This study has identified six human protein targets (PDB IDs 1O86, 4DJU, 1Q5K, 3G42, 4 M0E, and 7D8X) as potential candidates for AD therapy. Targeting or altering the activity of these proteins could potentially decelerate the advancement of AD.^51–53^ This approach is backed by numerous *in vivo* and *in vitro* studies, which have evaluated the effectiveness and safety of targeting these proteins as a strategy for AD treatment.^19,54,55^

The validation of the docking process was achieved by re-docking the co-crystallized ligands back into their respective active binding sites. The outcomes of this process, which include docking scores reflecting binding affinities, are depicted in Table 1 and Fig. S1.† The obtained root mean square deviations (RMSDs) were within an acceptable range, under 2 Å, as noted in ref. 56–58. Such validation lends credibility to the docking parameters used for predicting the binding orientations of new proaporphine alkaloids (cissamaline, cissamanine, cissamdine) with proteins associated with AD. Fig. S1† compares the superimposed conformations of the co-crystallized structures with the original docked ligands.

**Table 1:** Molecular docking scores (free binding energy; kcal mol^−1^) of the new proaporphine alkaloids (cissamaline, cissamanine, and cissamdine) and the co-crystallized ligands against potential therapeutic targets for Alzheimer's disease (AD)^a^

Compounds	Potential therapeutic targets for Alzheimer's disease					
	*1O86	*4DJU	*1Q5K	*3G42	*4 M0E	*7D8X
Cissamaline	−8.28	−7.53	−7.54	−9.27	−9.57	−7.47
Cissamanine	−8.87	−7.16	−7.70	−9.24	−9.87	−8.32
Cissamdine	−8.42	−8.51	−7.03	−9.92	−9.22	−8.16
*Co-crystalized ligand	−8.65	−6.92	−7.84	−9.83	−9.20	−10.19

a *1O86: the crystal structure of human angiotensin converting enzyme (ACE) in complex with the co-crystallized ligand (Lisinopril). *4DJU: the crystal structure of human β-site APP cleaving enzyme 1 (BACE1) in complex with the co-crystallized ligand (2-imino-3-methyl-5,5-diphenylimidazolidin-4-one). *1Q5K: the crystal structure of human glycogen synthase kinase-3 (GSK-3) in complex with the co-crystallized ligand (AR-A014418). *3G42: the crystal structure of human TNF-α converting enzyme (TACE) in complex with the co-crystallized ligand ((2*R*)-2-[(4-but-2-ynoxyphenyl)sulfonylamino]-3-(5-methyl-1*H*-indol-3-yl)propanoic acid). *4 M0E: the crystal structure of human acetylcholinesterase (AChE) in complex with the co-crystallized ligand (dihydrotanshinone I). *7D8X: structure of human gamma–secretase in complex with the co-crystallized ligand (L-685458).

The free binding energy and molecular interactions analysis, as depicted in Table 1 and Fig. S2–S7,† provide valuable insights into the binding capabilities of these compounds in comparison to the co-crystallized ligands for each respective protein target. The molecular docking scores, represented as free binding energies (in kcal mol^−1^), serve as a critical metric for evaluating the binding strength of the compounds to their respective protein targets. It is important to note that lower binding energies (more negative values) signify stronger binding interactions between ligands and proteins.

### Human angiotensin-converting enzyme (ACE)

ACE has been identified as a therapeutic target for the treatment of AD.^59^ ACE is an enzyme that plays a key role in the renin-angiotensin system, which regulates blood pressure and fluid balance in the body.^60^ Inhibition of ACE has been shown to have potential benefits in the treatment of AD, including reducing inflammation and promoting the clearance of β-amyloid protein.^59,61,62^ Preclinical and clinical studies have demonstrated that ACE inhibitors may improve cognitive function and reduce the risk of cognitive decline in individuals with AD.^62,63^ However, it is important to note that the optimal dosing and duration of treatment with ACE inhibitors for the treatment of AD has yet to be determined and requires further investigation.^59,61–63^

In previous studies, the active site of ACE was divided into three pockets: S1, S2, and S1′. S1 pocket contains the residues ALA354, GLU384, and TYR523, while S2 pocket consists of GLN281, HIS353, LYS511, HIS513, and TYR520. S1′ pocket includes the residue GLU162.^64–67^ ACE's active site also contains a zinc ion (Zn^2+^) that coordinates with HIS383, HIS387, and GLU411.^64–67^ Hydrogen bonding has been suggested to play a role in the binding of inhibitors to ACE.^64–67^

Lisinopril, the co-crystallized ligand with ACE, showcases a multifaceted interaction mechanism, highlighted by a free binding energy of −8.65 kcal mol^−1^ (Table 1). It engages in hydrogen bonding with residues HIS353 (2.76 Å, 3.24 Å), ALA354 (2.92 Å), TYR520 (2.56 Å), and TYR523 (2.77 Å), demonstrating its strong interaction (Fig. S1a†). Crucially, lisinopril forms a covalent bond with the zinc ion (ZN701) and establishes pi–sigma interaction with VAL518, alongside ionic interactions with GLU411 and LYS511, reinforcing its strong binding affinity and effectiveness as an ACE inhibitor.

Cissamaline's interaction within the ACE active site includes hydrogen bonds with ALA354 (2.51 Å) in the S1 pocket and GLN281 (1.98 Å), HIS353 (1.67 Å), and TYR520 (2.35 Å) in the S2 pocket, with a free binding energy of −8.28 kcal mol^−1^. Notably, its cyclohexa-2,5-dien-1-one group forms a covalent bond with the zinc ion (ZN701), a unique interaction observed with lisinopril, suggesting a potential mechanism as an ACE inhibitor^68^ (see Table 1 and Fig. S2b†).

Cissamanine formed hydrogen bonds with ALA354 (2.80 Å) in the S1 pocket and with HIS353 (2.26 Å), HIS513 (2.46 Å), and TYR520 (2.25 Å) in the S2 pocket, indicating a free binding energy of −8.87 kcal mol^−1^. Unlike lisinopril, it does not form a covalent bond with Zn^2+^, which could potentially affect its inhibitory activity. This distinction highlights cissamanine's reliance on alternative binding mechanisms for ACE inhibition, suggesting that while it shows strong potential, the absence of a covalent bond with Zn^2+^ might limit its effectiveness compared to inhibitors that engage directly with the metal ion^68^ (see Table 1 and Fig. S2c†).

Cissamdine interacted with ALA354 (3.54 Å) in the S1 pocket and GLU162 (2.71 Å) in the S1′ pocket, alongside GLU348 (2.09 Å), showcasing a distinct approach to ACE inhibition without coordinating with Zn^2+^, similarly to cissamanine, with a free binding energy of −8.42 kcal mol^−1^ (see Table 1 and Fig S2d†).

Among these compounds, cissamaline, through its mechanism of binding to the active site with its unique covalent bond interaction and hydrogen bonding, may exhibit the potential to inhibit ACE, closely mimicking the interaction pattern of lisinopril. However, further validation through empirical studies is necessary to confirm its inhibitory efficacy and explore the full scope of its potential as an ACE inhibitor.

### β-site APP cleaving enzyme 1 (BACE 1)

BACE1 plays a crucial role in the production of the β-amyloid peptide, which is a key component in the pathology of AD.^69–71^ BACE1 inhibition is a promising alternative for preventing the onset of AD, a hypothesis that has been known as the “amyloid cascade” since the 1990s.^69–73^ The cascade describes a series of neuropathological events that occur in sequence, starting with the accumulation of Aβ, which leads to the dysfunction of Tau proteins (which normally stabilize neuronal microtubules) and ultimately results in cell death through the aggregation of Tau proteins in the cell, compromising both dendrite and neuronal cell body functions.^69–73^ Many research teams are actively working on developing drugs to inhibit BACE1. However, BACE1 inhibitors is a formidable challenge because these inhibitors face difficulties in crossing the blood–brain barrier.^74–77^ Meanwhile, *in vitro* studies shown that BACE1 inhibitors are effective at preventing the formation of new amyloid plaques, but they are not effective at preventing the growth of existing plaques.^78–80^ This implies that the optimal utilization of BACE1 inhibitors may lie in their early administration, specifically to prevent plaque formation at the disease's onset.^78–80^

In a recent molecular docking study,^81^ 14 molecules were docked using Molex Virtual Docker to identify compounds that could be used as BACE1 inhibitors. Their interactions with the amino acids THR292, ASP93, ASP289, THR293, GLN134, ASN294, and THE133 were observed, and the inhibitory activity of these compounds was favoured by hydrogen bonds and hydrophobic interactions with these residues.

The binding of 2-imino-3-methyl-5,5-diphenylimidazolidin-4-one (co-crystallized in 4DJU.pdb) to the active binding site of BACE1 has been extensively studied and the results showed a binding affinity value of −6.92 kcal mol^−1^ (Table 1 and Fig. S1b†). The amino group at the imidazolidin-4-one moiety was found to play a crucial role in stabilizing the interaction between the ligand and BACE1 by forming two hydrogen bonds with ASP93 and ASP289 at distances of 2.67 Å and 2.40 Å, respectively. Two hydrophobic interactions were also observed between the diphenyl moieties of the ligand and LEU91 and ILE179 of BACE1.

The binding mode of cissamaline to the active binding site of BACE1 was investigated through computational docking simulations. The results revealed that cissamaline had a slightly more negative free binding energy compared to 2-imino-3-methyl-5,5-diphenylimidazolidin-4-one, with a value of −7.53 kcal mol^−1^ (Table 1). The 2-methoxy-6-methylcyclohexa-2,4-dien-1-one moiety of cissamaline was found to form two hydrogen bonds with the critical amino acids THR133 and GLN134 at distances of 1.93 Å and 2.54 Å, respectively. In addition, the carbonyl group at the cyclohexa-2,5-dien-1-one moiety formed one hydrogen bond with TRP137 at a distance of 1.81 Å. Furthermore, cissamaline also exhibited pi–sigma and pi–alkyl interactions with TYR132 (see Table 1 and Fig. S3b†).

The docking simulation of cissamanine revealed that it has a good fitting into the enzyme active site with a docking score of −7.16 kcal mol^−1^ which is almost similar to that of 2-imino-3-methyl-5,5-diphenylimidazolidin-4-one. As depicted in Fig. S3c,† cissamanine was observed to interact with the surrounding residues and formed two H-bonds: one between the carbonyl group at 6-hydroxy-2-methoxycyclohexa-2,4-dien-1-one moiety and TRP137 at distance 2.55 Å, and one between the carbonyl group at cyclohexa-2,5-dien-1-one moiety and PHE169 at distance 1.9 Å. Additionally, a hydrophobic interaction with LEU91 was also observed (see Table 1 and Fig. S3c†).

**Fig. 3:**
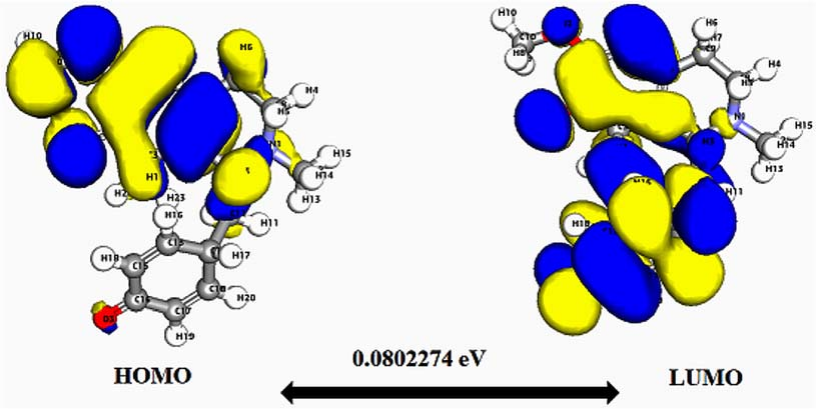
Depicts a graphical representation of the pictorial map showcasing the highest occupied molecular orbital (HOMO) and lowest unoccupied molecular orbital (LUMO) of the cissamaline compound.

Finally, the docking result of cissamdine revealed that it had a higher binding affinity (more negative) to BACE1 active binding site residues than 2-imino-3-methyl-5,5-diphenylimidazolidin-4-one, with an affinity value of −8.51 kcal mol^−1^. The amino group at the tetrahydropyridine moiety formed a strong hydrogen bond with the critical amino acid ASP93 at a distance of 1.62 Å. Furthermore, the carbonyl group at the cyclohexa-2,5-dien-1-one moiety formed two hydrogen bonds with SER97 and ASN98, at distances of 2.15 Å and 2.91 Å. Also, the hydroxyl group at 2-methoxycyclohexa-2,4-dien-1-ol created a hydrogen bond with PHE169 at 2.15 Å. Furthermore, cissamdine was also involved in binding with the enzyme through the formation of several hydrophobic interactions with VAL130, TRP137, and ILE179 (as depicted in Fig. 3d).

Considering the specific interactions detailed for the co-crystallized ligand, cissamdine's engagement with BACE1, marked by a strategic hydrogen bond with ASP93 and hydrophobic interaction with ILE179, may be promising. This nuanced approach, distinct from the control's interaction pattern, highlights cissamdine's potential as a new BACE1 inhibitor. However, further studies such as *in vitro* and *in vivo* experiments, are needed to validate these findings and to determine the effectiveness of these compounds as BACE1 inhibitors.

### Glycogen synthase kinase-3β (GSK-3β)

Glycogen synthase kinase-3 (GSK-3) is a protein that is responsible for the addition of phosphate molecules to serine and threonine residues, and it is encoded by two genes, GSK3α and GSK3β.^23,71,82,83^ The involvement of GSK-3β has been well established in neuropathological disorders such as amyloid deposition, gliosis, and tau hyperphosphorylation.^23,71,82,83^ Studies have shown that activation of the GSK3β gene contributes to the formation of beta-amyloid peptide (Aβ) and neurotic plaques, leading to an increase in Tau phosphorylation.^23,71,82–84^ GSK-3β consists of two major domains: an N-terminal β-strand domain extending from amino acid residues 25 to 138, and a C-terminal α-helical domain with residues 139 to 343.^82^ The interface between these two domains contains an ATP-binding site that is connected by a glycine-rich loop and hinge region.^82^ The ATP binding pocket involves residues LYS85, GLU97, ASP113, TYR134, VAL135, THR138, ASN186, LEU188, CYS199, and ASP200.^23,82,83^ In addition, GSK-3β′s catalytic activity is regulated by phosphorylation at the SER9 and TYR216 residues.^82,83^ Phosphorylation of the SER9 site inactivates GSK-3β, whereas phosphorylation at TYR216 within the activation loop increases its catalytic activity.^82,83^

The co-crystallized structure of GSK-3β with its inhibitor AR-A014418 (ref. 23) has revealed that the inhibitor binds to the hinge region and ATP pocket of the enzyme through formation of three hydrogen bonds: two with the residue VAL135 at distances of 2.68 Å and 2.78 Å, and one with TYR134 at distance of 2.41 Å. Furthermore, pi–cationic interactions are observed between the phenyl ring of AR-A014418 and the guanidinium group of ARG141. The structure is also stabilized by hydrophobic interactions with ALA83 and LEU188 (Fig. S1(c)†). From this structural analysis, it can be inferred that the inhibitor interferes with the activity of GSK-3β by binding to the ATP pocket and hinge region, disrupting the enzyme's ability to phosphorylate its substrates. This understanding of the binding interactions between GSK-3β and its inhibitors may aid in the discover new therapeutics for neurodegenerative disorders associated with GSK-3β activity.

The binding interactions between GSK-3β and the newly discovered proaporphine alkaloids (cissamaline, cissamanine, and cissamdine) were analysed and compared to the co-crystallized ligand AR-A014418 (as presented in Table 1 and Fig. S4†). The figure shows that all the new proaporphine alkaloids are able to interact with ATP binding site residues of GSK-3β through a combination of hydrogen bonds, pi–cationic interactions, and hydrophobic interactions.

As shown in Fig. S4b,† the analysis of cissamaline shows that it forms a hydrogen bond with the VAL135 residue, located in the ATP binding pocket, at a distance of 2.37 Å. This is similar to the binding of AR-A014418 to VAL135, however, the free binding energy (−7.54 kcal mol^−1^) is slightly weaker than that of AR-A014418 (−7.84 kcal mol^−1^). Further, cissamaline forms a map of hydrophobic interactions with several other residues located in the ATP binding pocket (VAL70, ALA83, LYS85, VAL110, LEU132, LEU188, and CYS199).

Similar to AR-A014418, cissamanine is shown to form hydrogen bonds with both TYR134 and VAL135, both located in the ATP binding pocket, at distances of 2.58 Å and 1.77 Å, respectively. In addition, the carbonyl group at the cyclohexa-2,5-dien-1-one moiety of cissamanine forms a hydrogen bond with the ASP200 residue, located in the ATP binding pocket, at a distance of 1.80 Å. The free binding energy of cissamanine (−7.70 kcal mol^−1^) is slightly weaker than that of AR-A014418. Cissamanine also forms a map of hydrophobic interactions with several other residues located in the ATP binding pocket (ILE62, VAL70, ALA83, VAL110, LEU132, LEU188, and CYS199) (Fig. S4c†). Finally, cissamdine is shown to form a hydrogen bond with the VAL135 residue, located in the ATP binding pocket, at a distance of 1.99 Å. Cissamdine also forms several hydrophobic interactions with several other residues located in the ATP binding pocket (VAL70, LYS85, VAL110, LEU132, and CYS199) (Fig. S4d†). The free binding energy of cissamdine (−7.03 kcal mol^−1^) is slightly weaker than that of AR-A014418.

Overall, the binding interactions and binding energies of the new proaporphine alkaloids were near to same as that of the co-crystallized ligand AR-A014418, with the exception that the new compounds have a little bit weaker free binding energy. Despite this, the new proaporphine alkaloids showed that they may have promising potential as inhibitors of GSK-3β, as they bind to the ATP binding pocket and hinge region similar to AR-A014418, which may disrupt the enzyme's ability to phosphorylate its substrates. Further studies are needed to determine the efficacy of these compounds as potential inhibitors of GSK-3β and their potential usefulness in treating neurodegenerative disorders associated with GSK-3β activity.

### TNF-α converting enzyme (TACE)

The TNF-α converting enzyme (TACE) is a serine and cysteine protease that plays a key role in the shedding of TNF-α, soluble TNF receptors, and fractalkine from the cell membrane.^85^ Studies have revealed that TACE has been associated with inflammatory diseases, such as Alzheimer's disease, as increased plasma TACE activity has been observed in subjects with mild cognitive impairment.^86–89^ Inhibition of TACE has the potential to mitigate TNFα levels and amyloid β deposition in Alzheimer's disease,^90^ while elevated cerebrospinal fluid (CSF) levels of TACE activity and soluble TNF receptors have been observed in patients with Alzheimer's disease.^87^

TACE is a multidomain metalloproteinase that processes tumor necrosis factor and a host of other proteins.^85,91,92^ The active binding site residue of TACE is a zinc binding motif and a P1′ side chain.^93^ Zinc plays a key role in the catalytic cycle of TACE.^94^ It is involved in the coordination of free cysteine residues in the pro-domain, preventing enzymatic activity,^95^ and binding of TNF-α results in partial oxidation followed by partial reduction of zinc.^94^ In addition, it is involved in the coordination of a free cysteine residue in the pro-domain, which prevents enzymatic activity and binding of TNF-α.^95^ Dysregulated activity of TACE is associated with inflammatory diseases and research is ongoing to harness the natural inhibitory domain to control TNFα production by regulating TACE activity.^96^

The X-ray structure of compound (2R)-2-[(4-but-2-ynoxyphenyl)sulfonylamino]-3-(5-methyl-1*H*-indol-3-yl)propanoic acid (co-crystallized ligand) with TACE^24^ was shown in Fig. S1(d).† Analysis of the structure revealed that the carboxylate moiety of the inhibitor is coordinated with the active site zinc ion, which is also coordinated with three histidine residues (HIS405, HIS409, and HIS415). In addition, the 5-methyl indole moiety of the co-crystallized ligand was found to be involved in hydrophobic interactions with HIS409, HIS415, and VAL353, and may also participate in a pi–sigma interaction with HIS409. The sulphonamide group of the 4-(prop-2-yn-1-yloxy)benzenesulfonamide moiety formed a hydrogen bond with LEU348 at a distance of 2.78 Å, and this moiety also formed hydrophobic interactions with LEU401, VAL402, HIS405, and ALA439. The coordination of the carboxylate moiety with the active site zinc ion is a key feature that contributes to the inhibition activity of the co-crystallized ligand.^24^ This binding blocks the access of substrate peptides to the active site, preventing the hydrolysis of the peptide bond and inhibiting TACE activity.^24^ The coordination complex formed between the carboxylate moiety and the zinc ion is highly stable, allowing the co-crystallized ligand to efficiently inhibit TACE. Furthermore, binding of the co-crystallized ligand to the zinc ion can induce conformational changes in the TACE enzyme, further contributing to its inhibition activity.^24^

In this study, the docking analysis of three new proaporphine alkaloids (cissamaline, cissamanine, and cissamdine) was performed to investigate their potential inhibitory activity against TACE (Fig. S5†). The results revealed that cissamaline, cissamanine, and cissamdine all formed interactions with the protein through hydrogen bonds and hydrophobic interactions (Fig. S5b–d†).

For instance, cissamaline formed a hydrogen bond with VAL440 at a distance of 2.98 Å, as well as a pi–sigma interaction with HIS405, and hydrophobic interactions with several residues including LEU348, VAL402, HIS405, HIS409, HIS415, and ALA439 (Fig. S5b†). Cissamanine formed two hydrogen bonds with TYR436 and VAL440 at distances of 1.80 Å and 3.14 Å, respectively and hydrophobic interactions with several residues including LEU348, VAL402, HIS405, HIS415, and ALA439 (Fig. S5c†). Cissamdine formed a hydrogen bond with GLU406 at a distance of 2.04 Å and another with PRO437 at a distance of 1.96 Å, and hydrophobic interactions with several residues including VAL402, HIS405, and ALA439 (Fig. S5d†). The free binding energy of the new proaporphine alkaloids was found to be slightly lower (less negative) than or the same as that of the co-crystallized ligand (−9.83 kcal mol^−1^). However, unlike the co-crystallized ligand, none of the new proaporphine alkaloids have interactions with the active site zinc ion. Lack of interaction with the active site zinc ion may affect the inhibitory activity of these phytocompounds compared to the co-crystallized ligand of 3G42.pdb.^97^

### Acetylcholinesterase (AChE)

Acetylcholinesterase (AChE) is a crucial enzyme that plays a vital role in regulating neurotransmission by degrading the neurotransmitter acetylcholine in the synapses of the nervous system.^3,4,25,30^ The AChE has been also shown to play a role in the formation of beta-amyloid (Aβ) aggregates in extracellular plaques in the brains of individuals with Alzheimer's disease (AD).^98–100^ As such, the inhibition of AChE may serve as a potential therapeutic strategy for preventing the formation of these toxic plaques.^98–100^ The enzyme possesses both catalytic and peripheral sites that are amenable to inhibition by various compounds.^3,4,25,30^

Drugs that are capable of crossing the blood–brain barrier and inhibiting AChE work by elevating the levels of acetylcholine, thereby potentiating its physiological effects.^3,4,25,30^ Reversible inhibitors of AChE are employed to compensate for decreased levels of endogenous acetylcholine in the treatment of Alzheimer's disease.^25^ More potent irreversible organophosphate (OP) inhibitors, on the other hand, completely block AChE activity by covalently modifying the catalytic SER203 site and have been utilized as chemical warfare nerve agents that cause death through paralysis as a result of the accumulation of acetylcholine in cholinergic synapses.^25^ A more comprehensive understanding of the mechanisms by which inhibitors interact with AChE may lead to the development of more efficacious therapeutics for treating cholinergic-related diseases or provide new strategies for protecting against OP poisoning.^25^

The structure of acetylcholinesterase (AChE) is composed of a central mixed β-sheet region surrounded by 15 α-helices.^101,102^ The catalytic anionic site (CAS) is located at the bottom of a narrow gorge and contains the esteratic site (SER203, GLU334, and HIS447) and anionic site (TRP86).^25,30,103^ In addition, there is another site known as the peripheral anionic site (PAS), which is composed of the residues TYR72, ASP74, TYR124, TRP286, and TYR341, and is located 20 Å away from the catalytic center.^25,30,102,103^ The PAS is characterized by the presence of an aromatic residue's ring, which creates 40% of the surface of the gorge and is located in a loop, allowing for greater conformational flexibility.^102^ In addition, the TRP86 residue forms a π-cation interaction with the quaternary nitrogen of the ACh along with PHE338.^25,30,102,103^ The PAS of AChE is known to act as an adhesion site for the non-amyloidogenic conformer of Aβ, leading to conformational changes that result in the production of amyloid fibrils.^102^ The TRP286 residue at the PAS mimics the response of the entire enzyme to amyloid formation. Furthermore, AChE–Aβ complexes have been shown to induce neurotoxicity and trigger more neurodegeneration than Aβ peptide alone.^102^ Therefore, designing an AChE inhibitor (AChEI) that blocks the PAS of the enzyme could prevent Aβ aggregation and enhance cholinergic transmission, making it a potential treatment for AD.^25,30,102,103^

Fig. S1e and S6† present the results of a 3D and 2D binding poses interactions analysis of the co-crystallized ligand dihydrotanshinone I and the new proaporphine alkaloids (cissamaline, cissamanine, and cissamdine), after docked to AChE structure (4M0E.PDB). The analysis includes information on the specific interactions between each compound and AChE, as well as the free binding energy (measured in kcal mol^−1^) of each interaction.

Starting with the co-crystallized inhibitor dihydrotanshinone I (Fig. S1(e)†), the analysis demonstrates that this compound forms hydrogen bonds with the PHE295 residue, located at the peripheral anionic site (PAS) of AChE, at distances of 1.65 Å and 2.88 Å. Furthermore, the aromatic ring in dihydrotanshinone I's structure engages in pi–sigma interactions with the PHE286 residue, also situated at the PAS. Additionally, dihydrotanshinone I establishes a network of hydrophobic interactions with TRP286, PHE297, TYR337, PHE338, and TYR341 residues at the PAS, resulting in a free binding energy of −9.20 kcal mol^−1^ (Table 1).

Moving on, cissamaline is shown to form an H-bond with the SER293 residue at a distance of 2.51 Å, a key component of the catalytic triad of AChE located at the catalytic anionic site (CAS). Additionally, cissamaline engages in a pi–sigma interaction with TRP286 and establishes several hydrophobic interactions with other residues at the PAS (TRP286, LEU289, VAL294, ARG296, PHE338, and TYR341) as depicted in Fig. S6b.† These interactions culminate in a free binding energy of −9.57 kcal mol^−1^ (Table 1).

Furthermore, cissamanine is shown to form three H-bonds with SER293, PHE295, and ARG296 at distances of 3.51 Å, 2.64 Å, and 2.41 Å, respectively, all located at the PAS of AChE. Additionally, it engages in a pi–sigma interaction with TRP286 and exhibits several hydrophobic interactions with TRP286, PHE338, and TYR341, also located at the PAS (Fig. S6c†). The free binding energy for this interaction is −9.87 kcal mol^−1^ (Table 1).

Finally, cissamdine is shown to form an H-bond with the TYR124 residue at a distance of 2.24 Å, located at the PAS, and two H-bonds with SER293 and PHE295 at distances of 2.24 Å and 2.20 Å, respectively, both situated at the PAS (Fig. S6d†). Additionally, it engages in hydrophobic interactions with TYR124, TRP286, TYR337, PHE338, and TYR341, all located at the PAS, resulting in a free binding energy of −9.22 kcal mol^−1^ (Table 1).

The analysis indicates that all three compounds engage AChE through H-bonds, hydrophobic, and/or pi–sigma interactions, mainly at the PAS. However, interactions and binding energies vary among the compounds, with dihydrotanshinone I and cissamanine displaying slightly stronger interactions with AChE than cissamaline and cissamdine. Thus, additional research is required to assess these compounds' effectiveness as AChE inhibitors and their potential role in Alzheimer's disease treatment.

### Gamma-secretase (GS)

Gamma-secretase (GS), an essential aspartyl protease embedded within the membrane, is known for its role in the selective cleavage of Type-I transmembrane (TM) proteins, influencing over a hundred distinct substrates with varying functions.^104^ This enzyme complex, characterized by its heterotetrameric composition, includes the presenilin homologues PS1 and PS2,^105^ nicastrin (Nct),^106^ anterior pharynx defective-1 (Aph-1),^107^ and presenilin enhancer-2 (Pen-2).^108^ Key among its substrates is the Amyloid Precursor Protein (APP)^109^ and the Notch receptors 1 to 4.^110^ The processing of APP by GS is of particular interest in the search for therapies that could alter the progression of Alzheimer's disease (AD) and similar conditions linked to GS.^20^ Agents aimed at GS are divided into inhibitors (GSIs), targeting the active site of the enzyme, and modulators (GSMs), which bind to a regulatory site, offering a refined method for therapeutic intervention.^20^

In investigating the inhibition of GS, a key enzymes implicated in AD, L-685458 emerges as a key model compound.^27,111^ As a transition state analog (TSA) and gamma-secretase inhibitor (GSI), it precisely targets the cleavage of the amyloid β-protein precursor by GS, achieving an IC50 of 17 nM.^27,111^ It exhibits exceptional selectivity, being over 50 to 100 times more potent against GS than other aspartyl proteases. Additionally, L-685458 effectively inhibits the cleavage of significant substrates, such as APP-C99 and Notch-100, as indicated by IC50 values of 301.3 nM and 351.3 nM, respectively.^27,111^

Crucially, the co-crystallization of L-685458 with the GS complex, documented in the Protein Data Bank under entry 7D8X,^27^ offers valuable structural insights into its inhibitory action. Understanding the molecular interactions of L-685458 could facilitate the exploration of the potential inhibitory effects of new compounds, such as proaporphine alkaloids (cissamaline, cissamanine, cissamdine), against GS. This may offer a strategic avenue for discovering novel therapeutic approaches.

L-685458 distinguishes itself with a free binding energy of −10.19 kcal mol^−1^, signaling a strong affinity for GS (Table 1). This ligand is characterized by its hydrogen bonding with ASP257 (2.63 Å), LYS380 (2.87 Å and again at 2.96 Å), GLY382 (2.84 Å), GLY384 (2.83 Å), and LEU432 (2.54 Å and 2.91 Å), along with hydrophobic interactions with LEU268, VAL272, LEU286, LEU422, and LEU425 (Fig. S1f†). These comprehensive interactions underpin a tightly bound complex, highlighting the specificity and stability conferred by both hydrogen bonds and hydrophobic contacts.

Cissamaline, exhibiting a free binding energy of −7.47 kcal mol^−1^ (Table 1), indicates a weaker affinity towards GS. Its interaction profile, featuring a single hydrogen bond with LEU432 (2.31 Å) and hydrophobic contacts with VAL261, ILE287, and ALA434 (Fig. S7b†), suggests a limited engagement with the enzyme's active site, potentially reflecting lower inhibitory potency. The reduced hydrogen bonding, especially with critical residues essential for the strong inhibition observed with L-685458, underscores a significant gap in interaction specificity and binding affinity.

Cissamanine shows a moderate free binding energy of −8.32 kcal mol^−1^ (Table 1) and interacts through hydrogen bonds with LYS380 (3.19 Å), ASP385 (2.26 Å), and ALA434 (2.92 Å and 2.93 Å), along with hydrophobic contacts with VAL261 and ALA431 (Fig. S7c†). Despite a more extensive interaction pattern than cissamaline, it falls short of mimicking L-685458's dense network of hydrogen and hydrophobic interactions, reflecting a diminished inhibitory potential.

Cissamdine, exhibits a free binding energy of −8.16 kcal mol^−1^ (Table 1). It forms hydrogen bonds with LEU286 (2.08 Å), LEU383 (3.16 Å), and ASP385 (2.24 Å), complemented by hydrophobic interactions with VAL261, LEU268, and ALA431 (Fig. S7d†). This configuration, while representing a step towards a more engaged interaction with GS, still does not achieve the comprehensive and potent binding observed with L-685458. The detailed engagement with key residues through both hydrogen bonding and hydrophobic interactions, although improved, does not fully replicate the intricate molecular interface established by the co-crystallized ligand.

Overall, the proaporphine alkaloids—cissamaline, cissamanine, and cissamdine—despite their interactions with gamma-secretase, may not achieve the potency of L-685458, the benchmark co-crystallized ligand. Their current levels of molecular interaction do not qualify them as potent inhibitors of gamma-secretase. However, these compounds possess potential as foundational scaffolds for further development.

In general, the molecular docking analysis indicates that cissamaline, cissamanine, and cissamdine exhibit potential as inhibitors against specific Alzheimer's disease targets. This foundational study prompts further exploration of their inhibitory mechanisms. Future research, incorporating Density Functional Theory (DFT) and Molecular Electrostatic Potential (MEP) analyses, is essential. These advanced computational techniques will allow for a more detailed understanding of the compounds' electronic properties, charge distributions, and interaction dynamics, paving the way for their potential development into therapeutic agents for Alzheimer's disease.

### Molecular reactivity analysis

#### Density function theory

Density Functional Theory (DFT) calculations for cissamaline, cissamanine, and cissamdine reveal distinct electronic and energetic characteristics that are crucial for understanding their chemical behavior and potential applications. The differences in total energy and binding energy across these compounds suggest variations in their molecular stability and reactivity (Table S3†). Cissamaline, with a total energy of −1009.32 and a binding energy of −8.89957, suggests a stable but reactive molecular structure. In contrast, cissamanine, with a slightly lower total energy of −1045.05 and binding energy of −8.52111, might exhibit a different reactivity profile. Cissamdine stands out with a total energy of −971.544 and binding energy of −8.58034, indicating unique stability characteristics.

**Fig. 4:**
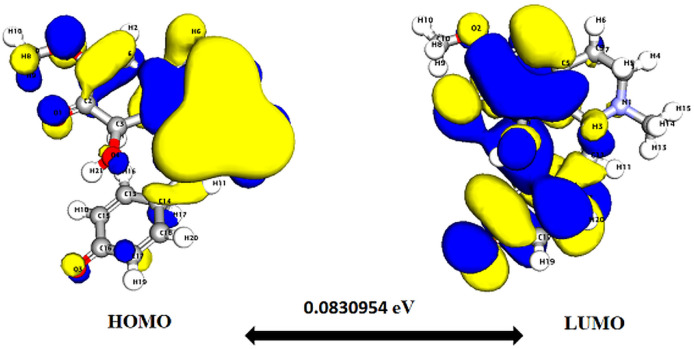
Depicts a graphical representation of the pictorial map showcasing the highest occupied molecular orbital (HOMO) and lowest unoccupied molecular orbital (LUMO) of the cissamanine compound.

**Fig. 5:**
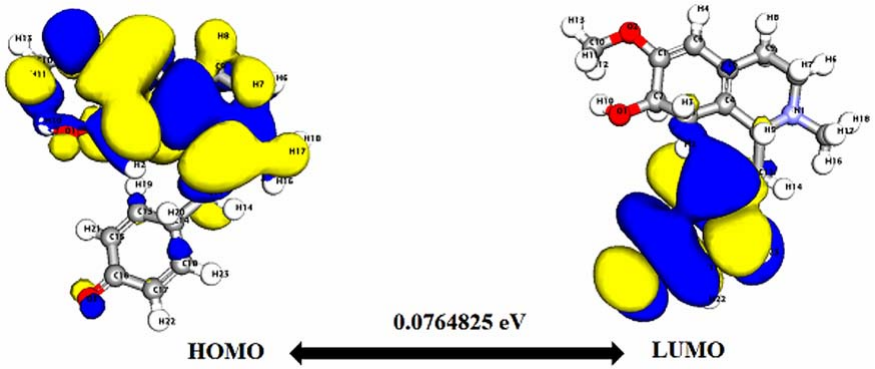
Depicts a graphical representation of the pictorial map showcasing the highest occupied molecular orbital (HOMO) and lowest unoccupied molecular orbital (LUMO) of the cissamdine compound.

Significantly, the HOMO–LUMO gap is a crucial factor in determining electronic properties. Cissamaline exhibits a HOMO energy of −0.182999 and a LUMO energy of −0.102772, resulting in a band gap of 0.0802274 (Fig. 3). This band gap indicates a balance between stability and reactivity, suitable for applications requiring moderate electron transfer. Cissamanine, with a HOMO energy of −0.184059 and a LUMO energy of −0.100964, has a slightly larger band gap of 0.0830954 (Fig. 4), suggesting lower reactivity. Conversely, cissamdine's smaller band gap of 0.0764825 (Fig. 5), derived from its HOMO energy of −0.171744 and LUMO energy of −0.0952612, points to a higher reactivity and ease of electron transition, making it potentially more suitable for applications involving rapid electron exchange.

The dipole moments of these compounds highlight differences in their polarity. Cissamaline's dipole moment of 2.95921 and cissamanine's of 2.97755 indicate stronger interactions in polar environments, affecting solubility and reactivity, compared to Cissamdine with a lower dipole moment of 1.86533. Furthermore, the hardness and softness values, with Cissamdine showing a lower hardness (0.0382) and higher softness (26.15), suggest its more flexible and ionizable nature, whereas cissamaline and cissamanine, with higher hardness values (0.0406 and 0.0415, respectively) and lower softness values (24.61 and 24.07, respectively), might resist deformation and ionization more effectively.

Lastly, the electrophilicity index (*ω*) provides insights into the compounds' tendencies to accept electrons. Cissamaline's notably high electrophilicity (137.11) suggests it is more likely to engage in electrophilic reactions, a property that could be harnessed in synthetic chemistry applications. The electronegativity (*χ*) values, relatively close among the compounds, hint at comparable electron affinities, which is essential in predicting their behavior in chemical reactions.

Overall, the detailed DFT analysis underscores the unique electronic and energetic characteristics of cissamdine, cissamaline, and cissamanine. These insights inform their potential applications in various fields, such as pharmaceuticals, material science, and synthetic chemistry, where specific properties like reactivity, polarity, mechanical resistance, and electron affinity play crucial roles. The nuanced understanding of these properties facilitates targeted compound design, optimizing their use in specialized applications.

### Molecular electrostatic calculations

**Fig. 6:**
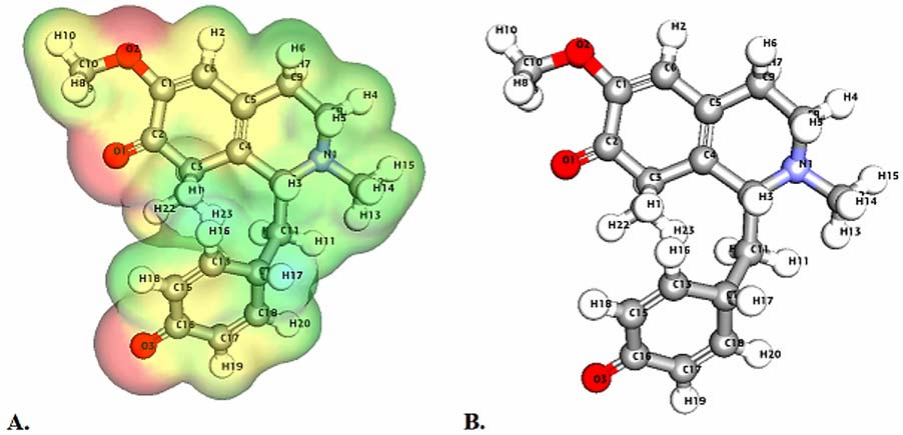
Illustrates the Three-dimensional Molecular Electrostatic Potential (3D-MEP) of the cissamaline complex, with the areas of highest positive potential depicted in deep blue and the regions of most negative potential in deep yellow (Panel A). Furthermore, Panel B displays the optimized structure of the cissamaline complex.

**Fig. 7:**
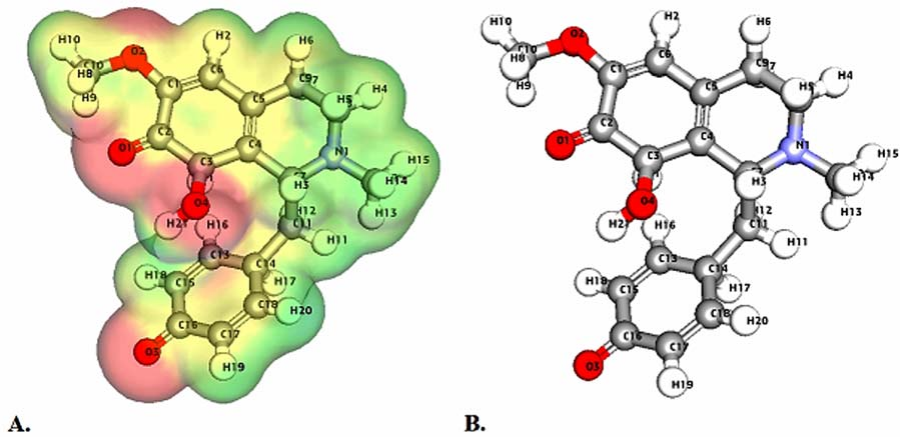
Illustrates the Three-dimensional Molecular Electrostatic Potential (3D-MEP) of the cissamanine complex, with the areas of highest positive potential depicted in deep blue and the regions of most negative potential in deep yellow (Panel A). Furthermore, Panel B displays the optimized structure of the cissamanine complex.

**Fig. 8:**
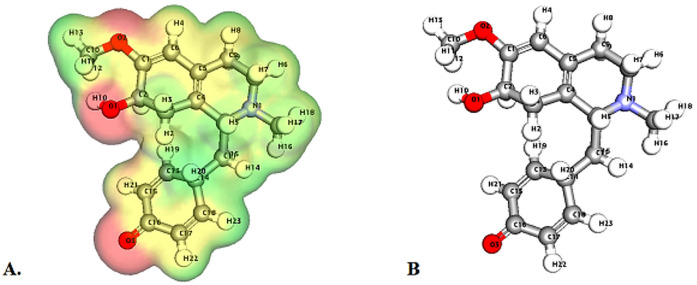
Illustrates the Three-dimensional Molecular Electrostatic Potential (3D-MEP) of the Cissamdine complex, with the areas of highest positive potential depicted in deep blue and the regions of most negative potential in deep yellow (Panel A). Furthermore, Panel B displays the optimized structure of the cissamdine complex.

Molecular Electrostatic Potential (MEP) analyses for the compounds cissamaline, cissamanine, and cissamdine, as illustrated in Fig. 6–8, offer profound insights into their chemical reactivity and interaction potential. These MEP analyses, crucial for identifying regions prone to electrophilic attacks, nucleophilic reactivity, and hydrogen bonding, reveal that all three compounds exhibit significant electrostatic characteristics governed by the presence of positively charged nitrogen atoms and negatively charged oxygen atoms.

In cissamaline (Fig. 6), the nitrogen atom's lone pair of electrons contributes to a negative electrostatic potential, indicating nucleophilic capabilities, while the oxygen atoms' polarization results in positive potentials, highlighting its role as a hydrogen bond acceptor. Similarly, cissamanine's MEP features (Fig. 7), with negative potential around the nitrogen and positive around the oxygen atoms, suggest its proficiency in nucleophilic reactions and hydrogen bonding. Cissamdine follows this trend (Fig. 8), with its MEP analysis suggesting a similar chemical behavior characterized by potential nucleophilic activity and hydrogen bond acceptives.

Integrating the findings from both DFT and MEP analyses, we observe a comprehensive profile of the electronic, energetic, and electrostatic properties of cissamaline, cissamanine, and cissamdine. The DFT analysis, revealing critical data on total and binding energies, HOMO–LUMO gaps, and other electronic parameters, along with the MEP study highlighting areas susceptible to electrophilic and nucleophilic attacks, collectively provides a deep understanding of these compounds' chemical behaviors. These properties, particularly the unique electronic configurations and reactivity profiles suggested by the DFT analysis, combined with their MEP-derived potential for specific biochemical interactions, underscore the promise of cissamaline, cissamanine, and cissamdine in AD treatment.

The theoretical predictions suggest these compounds could interact beneficially with biological targets implicated in AD, but the transition from theoretical efficacy to practical application requires empirical evidence. Therefore, further *in vitro* and *in vivo* studies are essential to confirm the therapeutic potential of these compounds in AD treatment. Such studies will not only validate their effectiveness and safety but also provide crucial insights into their mechanism of action, paving the way for their potential use as new agents in combating AD.

## Conclusions

This study marks a significant stride in exploring the therapeutic potential of *Cissampelos capensis* L.f., particularly its proaporphine alkaloids—cissamaline, cissamanine, and cissamdine—for Alzheimer's Disease (AD) treatment. Our in-depth *in silico* analysis revealed that these alkaloids exhibit favorable pharmacokinetic profiles and the ability to penetrate the blood–brain barrier, crucial for CNS-targeted therapies. Molecular docking studies indicate that cissamaline, cissamanine, and cissamdine interact with key AD-associated proteins—demonstrating potential as inhibitors of Angiotensin-Converting Enzyme (ACE) and β-site APP cleaving enzyme 1 (BACE1) and exhibiting inhibitory characteristics against Glycogen Synthase Kinase-3β (GSK-3β) and Acetylcholinesterase (AChE). These interactions are comparable to, or in some aspects slightly less potent than, those observed with established AD drugs, highlighting their potential as new therapeutic agents for Alzheimer's disease. The incorporation of Density Functional Theory (DFT) and Molecular Electrostatic Potential (MEP) analyses provided a deeper understanding of the alkaloids' electronic and energetic characteristics. These analyses revealed unique electronic properties, including differences in total energy, binding energy, and HOMO–LUMO gaps, which are indicative of their molecular stability and reactivity. The MEP visualizations further illustrated their electrostatic characteristics, aiding in the prediction of biochemical interactions. These findings collectively illuminate the potential of cissamaline, cissamanine, and cissamdine as novel candidates for AD therapy. Their unique electronic, energetic, and binding properties position them as promising agents in the quest for effective AD treatments. However, while these *in silico* results are encouraging, they underscore the necessity for subsequent empirical research. Further *in vitro* and *in vivo* investigations are essential to validate these theoretical predictions and to elucidate the precise mechanisms through which these alkaloids exert their therapeutic effects. The transition from theoretical efficacy to practical application remains crucial, paving the way for potential new treatments in the battle against AD. To build on this foundation, future research will focus on molecular dynamics simulations to assess the dynamic interactions and stability of these compounds with AD-related targets, ensuring a comprehensive understanding of their therapeutic potential.

## Author contributions

Conceptualization (M. B. A.); methodology (A. D., A. T., M. G. A., M. B. A.); software (Gaussian software for DFT and MESP calculations: A. D., A. T.; additional contributions: D. F., B. H. A.); validation (M. B. A., M. G. A., A. D., A. T.); formal analysis (A. D., A. T., M. G. A.); investigation (M. D.); resources (A. D., A. T., D. F., B. H. A.); data curation (M. B. A., M. G. A.); writing – original draft (M. B. A., M. G. A., A. D., A. T.); writing – review & editing (D. F., M. D.); visualization (A. D., A. T., M. G. A.); supervision (M. B. A.); project administration (M. B. A.); funding acquisition (M. B. A.). All authors have read and agreed to the published version of the manuscript. The specific use of Gaussian software for DFT and MESP calculations by A. D. and A. T. was a critical contribution to the study's computational analysis.

## Conflicts of interest

There are no conflicts to declare.

## Supplementary Material

RA-014-D4RA01070A-s001
