# Supplementary material for: ADME profiling, molecular docking, DFT, and MEP analysis reveal cissamaline, cissamanine, and cissamdine from Cissampelos capensis L.f. as potential anti-Alzheimer's agents
Source: RSC Adv. 2024 Mar 25;14(14):9878–91. doi: 10.1039/d4ra01070a (PMC10961956; doi:10.1039/d4ra01070a)
Supplement: RA-014-D4RA01070A-s001 [file RA-014-D4RA01070A-s001.pdf]

Electronic Supplementary Material (ESI) for RSC Advances.
This journal is © The Royal Society of Chemistry 2024

Supplementary Materials

ADME Profiling, Molecular Docking, DFT, and MEP Analysis Reveal Cissamaline, Cissamanine, and Cissamdine from *Cissampelos capensis* L.f. as Potential Anti-Alzheimer Agents

Maram B. Alhawarri*[a], Mohammad G. Al-Thiabat[b], Amit Dubey[c,d], Aisha Tufail[d], Dania Fouad[e], Bilal Harieth Alrimawi[f], and Mohamad Dayoob[g]

[a]Department of Pharmacy, Faculty of Pharmacy, Jadara University, P.O.Box 733, Irbid 21110, Jordan. m.hawarri@jadara.edu.jo

[b]School of Pharmaceutical Sciences, Universiti Sains Malaysia, Gelugor 11800, Penang, Malaysia. Mohd.althiabat@gmail.com

[c]Department of Pharmacology, Saveetha Dental College and Hospital, Saveetha Institute of Medical and Technical Sciences, Chennai-600077, Tamil Nadu, India. ameetbioinfo@gmail.com

[d]Computational Chemistry and Drug Discovery Division, Quanta Calculus, Greater Noida-201310, Uttar Pradesh, India. aishatufailansari@gmail.com

[e]Faculty of Dentistry, Ibn Sina University for Medical and Pharmaceutical Sciences, Baghdad, Iraq. daniaalsafar2009@gmail.com

[f]Michael Sayegh Faculty of Pharmacy, Aqaba University of Technology, Aqaba, Jordan. brimawi@aut.edu.jo

[g]Faculty of Pharmacy, MAHSA University, Jenjarom, Malaysia. mohamaddayoob@mahsa.edu.my

***Corresponding authors**
Dr. Maram B. Alhawarri
Department of Pharmacy
Faculty of Pharmacy
Jadara University
Irbid 21110, Jordan
E-mail: m.hawarri@jadara.edu.jo

Table S1. Grid box coordinates of protein-ligand interactions as determined by co-crystallized inhibitors.

Crystal structure	Grid box size	Active binding sites coordinate (xyz-coordinates)		
		X	Y	Z
ACE-1O86	40*40*40	40.553	32.798	47.286
BACE1-4DJU	40*40*40	21.029	11.195	21.798
GSK3-1Q5K	40*40*40	23.148	22.189	9.398
TACE-3G42	40*40*40	52.568	33.061	104.89
AChE-4M0E	40*40*40	-17.171	-42.504	25.612
GS-7D8X	40*40*40	164.22	174.11	148.43

Table S2. Predicted cytochrome (CYP) enzyme inhibition properties of cissamaline, cissamanine, and cissamdine utilizing the SwissADME web service.

Property	Model Name	Predicted Value		
		Cissamaline	Cissamanine	Cissamdine
**Metabolism**	CYP1A2 substrate	No	No	No
	CYP2C19 substrate	No	No	No
	CYP2C9 substrate	No	No	No
	CYP2D6 substrate	No	No	No
	CYP3A4 inhibitor	No	No	No

Table S3. Density Function Theory calculations with other descriptors.

Name	Total energy	Binding Energy	HOMO Energy	LUMO Energy	Band Gap Energy	Dipole moment	Hardness (η)	Softness (S)	Electro-philicity (ω)	Electroneg-ativity (χ)
**Cissamdine**	-971.544	-8.58034	-0.171744	-0.0952612	0.0764825	1.86533	0.0382	26.15	46.00	0.1335
**Cissamaline**	-1,009.32	-8.89957	-0.182999	-0.102772	0.0802274	2.95921	0.0406	24.61	137.11	0.1429
**Cissamanine**	-1,045.05	-8.52111	-0.184059	-0.100964	0.0830954	2.97755	0.0415	24.07	108.75	0.1425

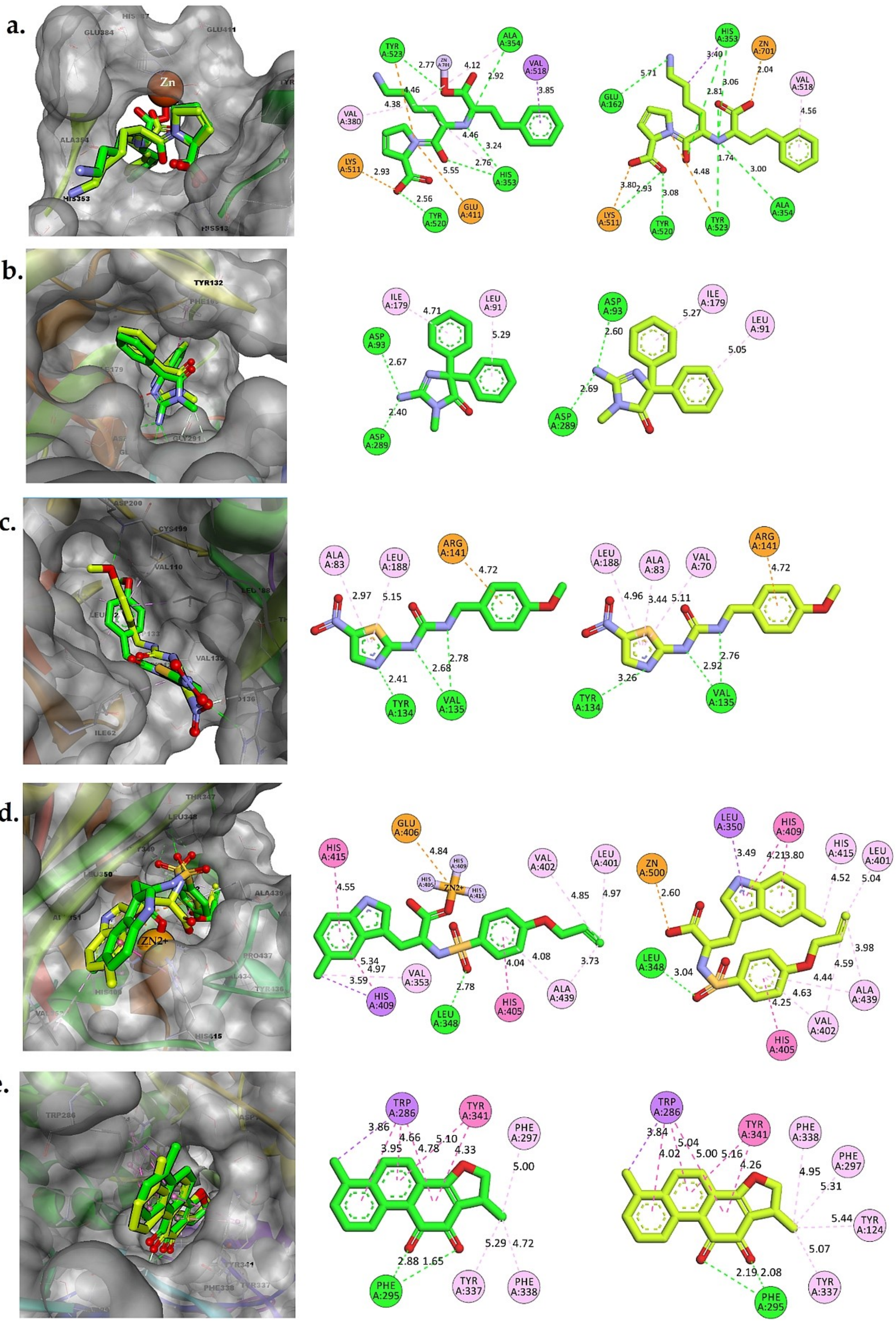
a.
b.
c.
d.
e.
Zn
TYR A:523
ALA A:354
VAL A:518
VAL A:380
LYS A:511
HIS A:353
TYR A:520
GLU A:411
HIS A:353
ZN A:701
GLU A:162
VAL A:518
LYS A:511
TYR A:520
TYR A:523
ALA A:354
ILE A:179
LEU A:91
ASP A:93
ASP A:289
ASP A:93
ILE A:179
LEU A:91
ASP A:289
ALA A:83
LEU A:188
ARG A:141
TYR A:134
VAL A:135
LEU A:188
ALA A:83
VAL A:70
ARG A:141
TYR A:134
VAL A:135
HIS A:415
GLU A:406
VAL A:402
LEU A:401
VAL A:353
HIS A:409
LEU A:348
HIS A:405
ALA A:439
LEU A:350
HIS A:409
ZN A:500
HIS A:415
LEU A:401
LEU A:348
ALA A:439
VAL A:402
HIS A:405
TRP A:286
TYR A:341
PHE A:297
PHE A:295
TYR A:337
PHE A:338
TRP A:286
TYR A:341
PHE A:338
PHE A:297
TYR A:124
TYR A:337
PHE A:295

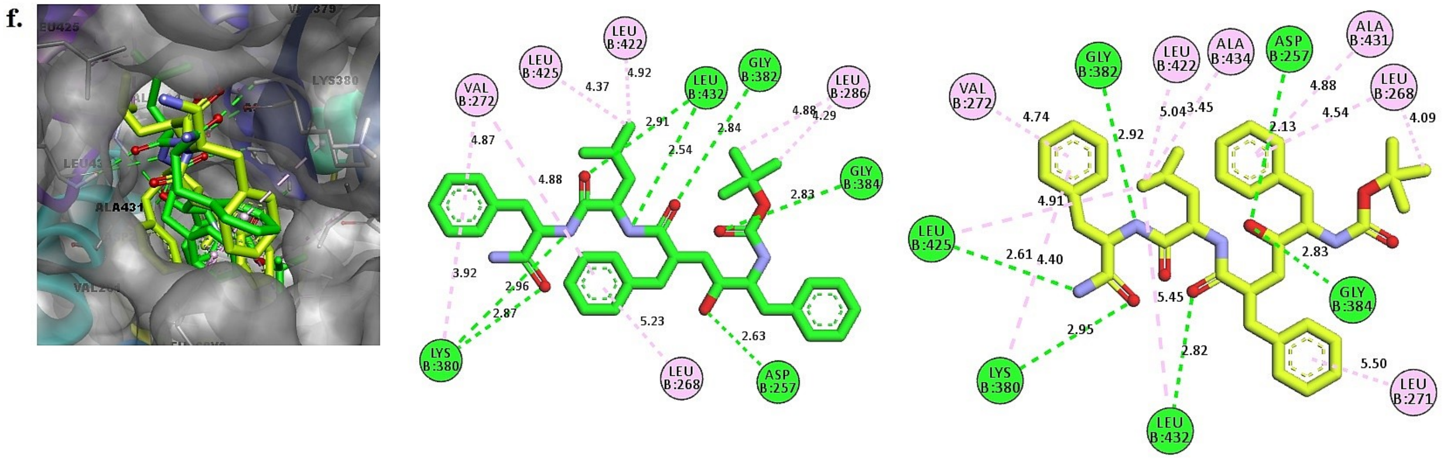

Figure S1. Superimposition and 2D interactions analysis of the co-crystallized ligand (green C, red O, and blue N) and re-docked ligand (lime C, red O, and blue N). (a) The crystal structure of human angiotensin converting enzyme (ACE) in complex with the co-crystallized ligand (Lisinopril) (1O86.pdb). (b) The crystal structure of human β-site APP cleaving enzyme 1 (BACE1) in complex with the co-crystallized ligand (2-imino-3-methyl-5,5-diphenylimidazolidin-4-one) (4DJU.pdb). (c) The crystal structure of human glycogen synthase kinase-3 (GSK-3) in complex with the co-crystallized ligand (AR-A014418) (1Q5K.pdb). (d) The crystal structure of human TNF-α converting enzyme (TACE) in complex with the co-crystallized ligand (2R)-2-[(4-but-2-ynoxyphenyl)sulfonylamino]-3-(5-methyl-1H-indol-3-yl)propanoic acid) (3G42.pdb). (e) The crystal structure of human acetylcholinesterase (AChE) in complex with the co-crystallized ligand (Dihydrotanshinone I) (4M0E.pdb). (f) The crystal structure of the human gamma-secretase (GS) complex in complex with the co-crystallized ligand (L-685458) (PDB ID: 7D8X).

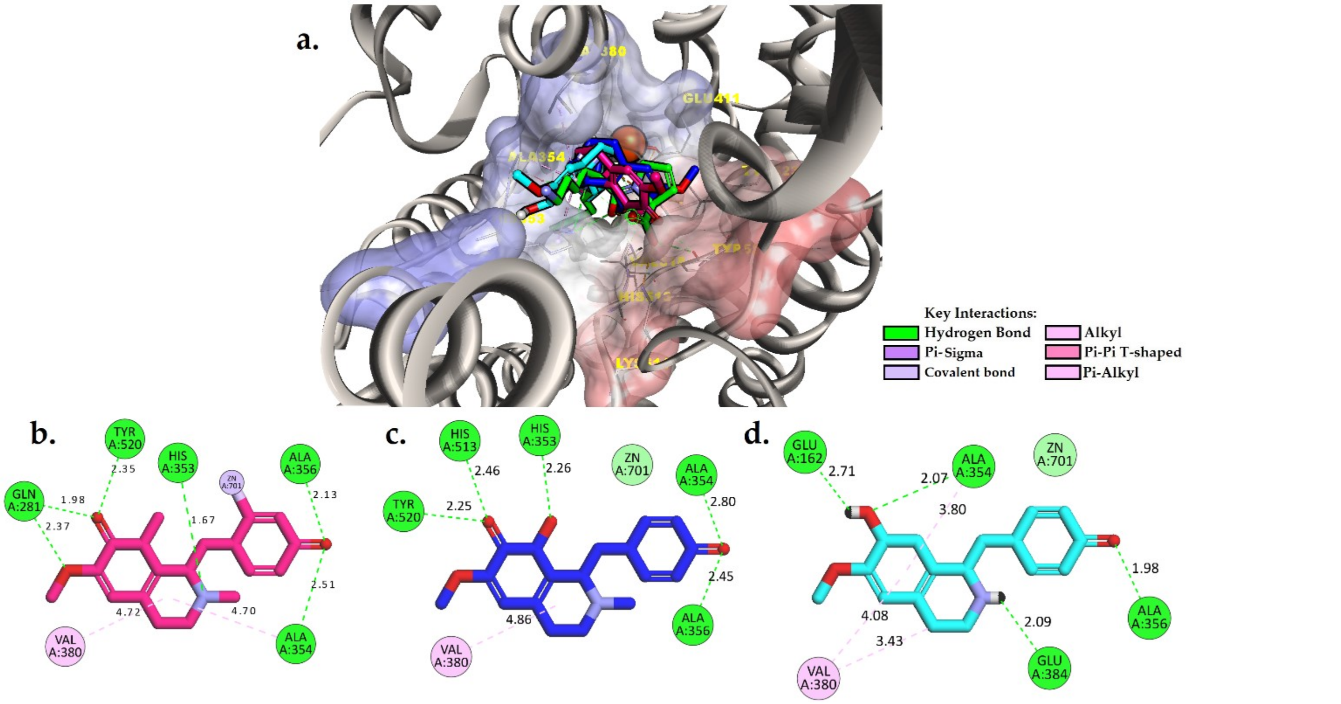

Figure S2. 3D binding pose interactions between lisinopril (green C, red O, pale blue N), cissamaline (pink C, red O, pale blue N), cissamanine (blue C, red O, pale blue N), and cissamdine (cyan C, red O, pale blue N) and angiotensin converting enzyme (ACE) (a). 2D interaction analysis of cissamaline (b), cissamanine (c), and cissamdine (d) docked in the active binding site of ACE, represented by a solid surface rendering with pKa color-coding. ACE (PDB ID: 1O86) is shown as a gray ribbon.

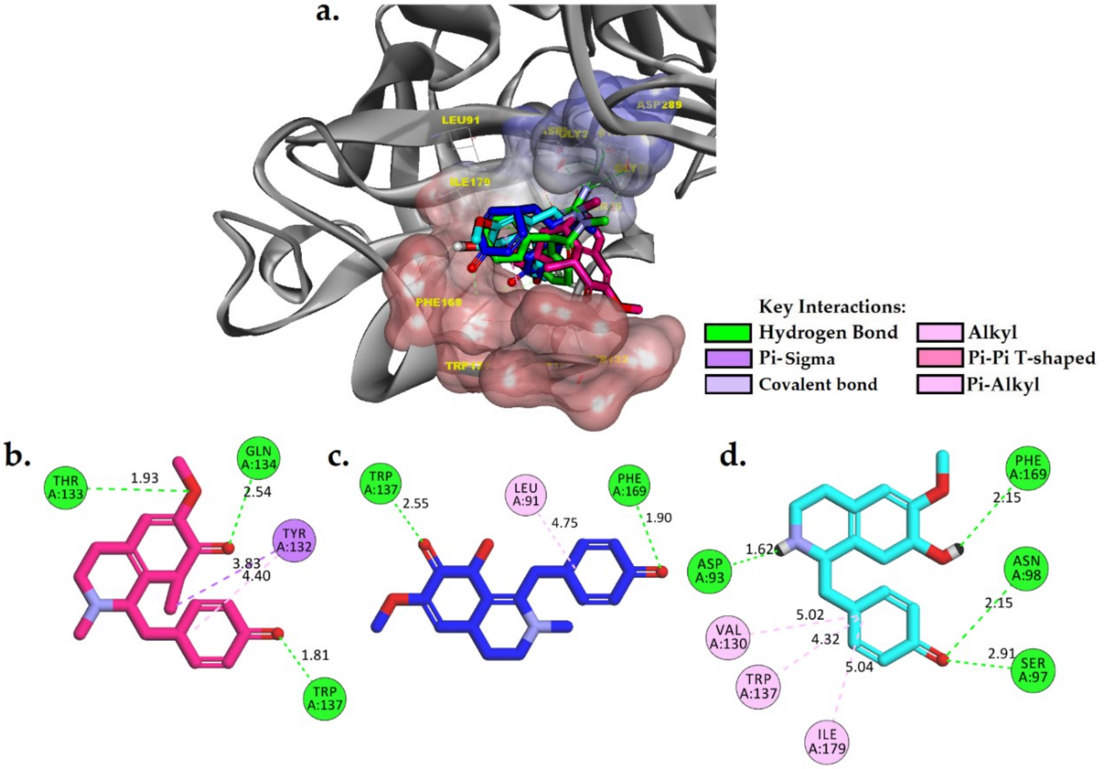

Figure S3. 3D binding poses interactions between 2-imino-3-methyl-5,5-diphenylimidazolidin-4-one (green C, red O, pale blue N), cissamaline (pink C, red O, pale blue N), cissamanine (blue C, red O, pale blue N), and cissamdine (cyan C, red O, pale blue N) and β-site APP cleaving enzyme 1 (BACE1) (a). 2D interaction analysis of cissamaline (b), cissamanine (c), and cissamdine (d) docked in the active binding site of BACE1, represented by a solid surface rendering with pKa color-coding. BACE1 (PDB ID: 4DJU) is shown as a gray ribbon.

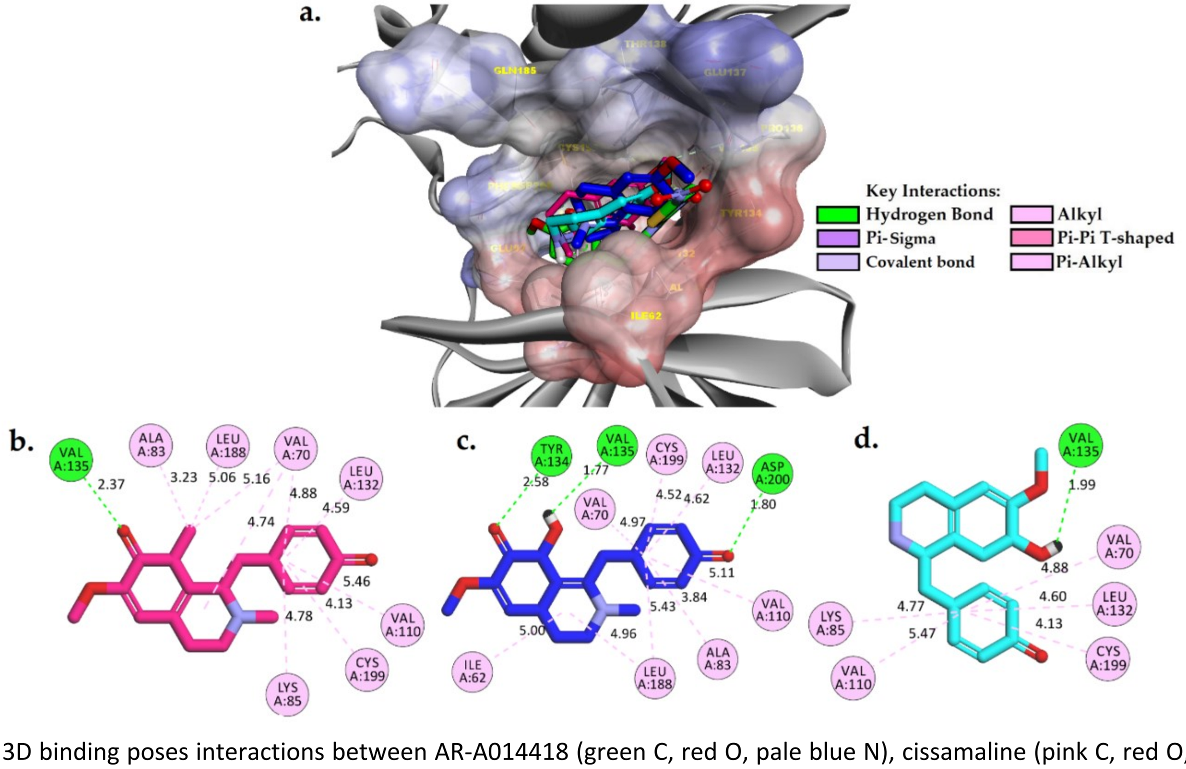

Figure S4. 3D binding poses interactions between AR-A014418 (green C, red O, pale blue N), cissamaline (pink C, red O, pale blue N), cissamanine (blue C, red O, pale blue N), and cissamdine (cyan C, red O, pale blue N) and Glycogen Synthase Kinase-3β (GSK-3β) (a). 2D interaction analysis of cissamaline (b), cissamanine (c), and cissamdine (d) docked in the active binding site of GSK-3β, represented by a solid surface rendering with pKa color-coding. BACE1 (PDB ID: 4DJU) is shown as a gray ribbon.

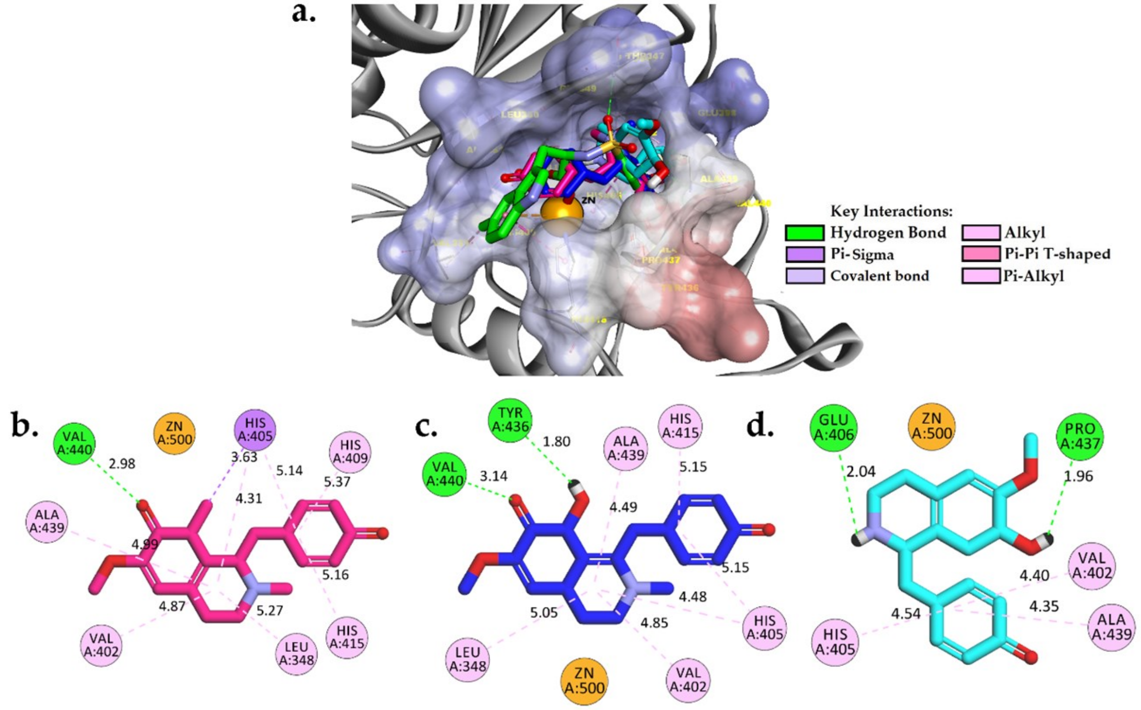

Figure S5. 3D binding poses interactions between (2R)-2-[(4-but-2-ynoxyphenyl)sulfonylamino]-3-(5-methyl-1H-indol-3-yl)propanoic acid (green C, red O, pale blue N), cissamaline (pink C, red O, pale blue N), cissamanine (blue C, red O, pale blue N), and cissamdine (cyan C, red O, pale blue N) and TACE (a). 2D interaction analysis of cissamaline (b), cissamanine (c), and cissamdine (d) docked in the active binding site of TACE, represented by a solid surface rendering with pKa color-coding. TACE (PDB ID: 3G42) is shown as a gray ribbon.

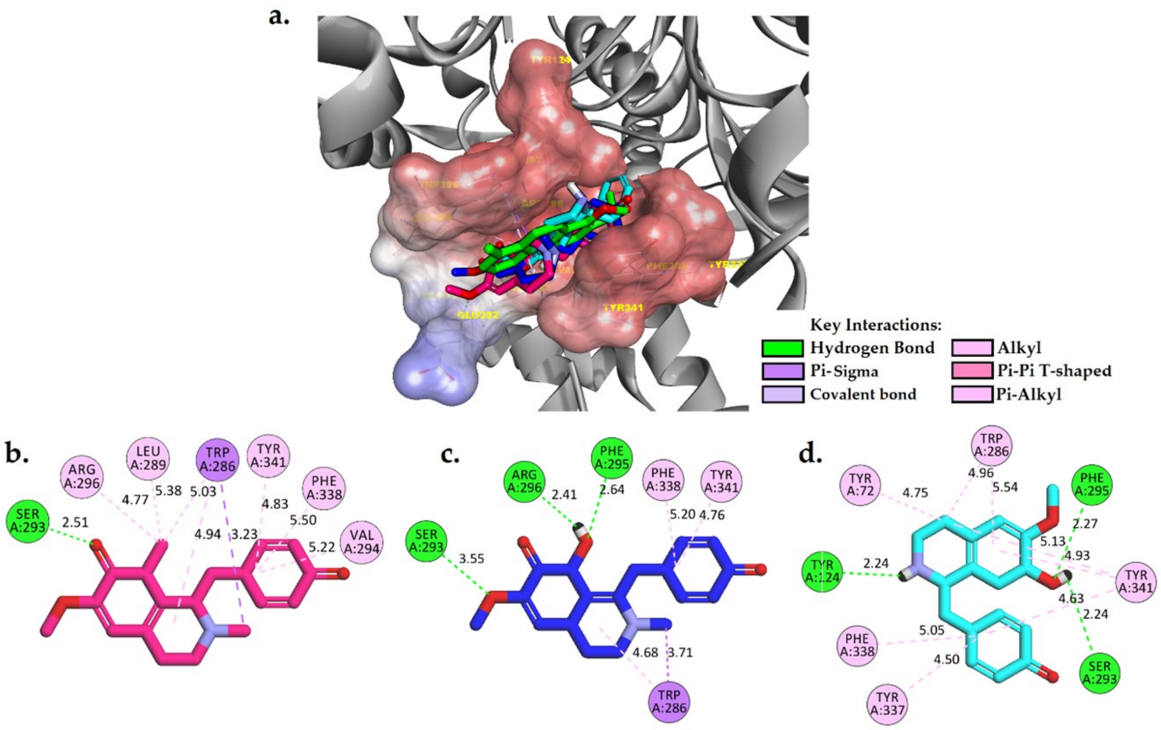

Figure S6. 3D binding poses interactions between Dihydrotanshinone I (green C, red O, pale blue N), cissamaline (pink C, red O, pale blue N), cissamanine (blue C, red O, pale blue N), and cissamdine (cyan C, red O, pale blue N) and AChE (a). 2D interaction analysis of cissamaline (b), cissamanine (c), and cissamdine (d) docked in the active binding site of AChE, represented by a solid surface

rendering with pKa color-coding. AChE (PDB ID: 4M0E) is shown as a gray ribbon.

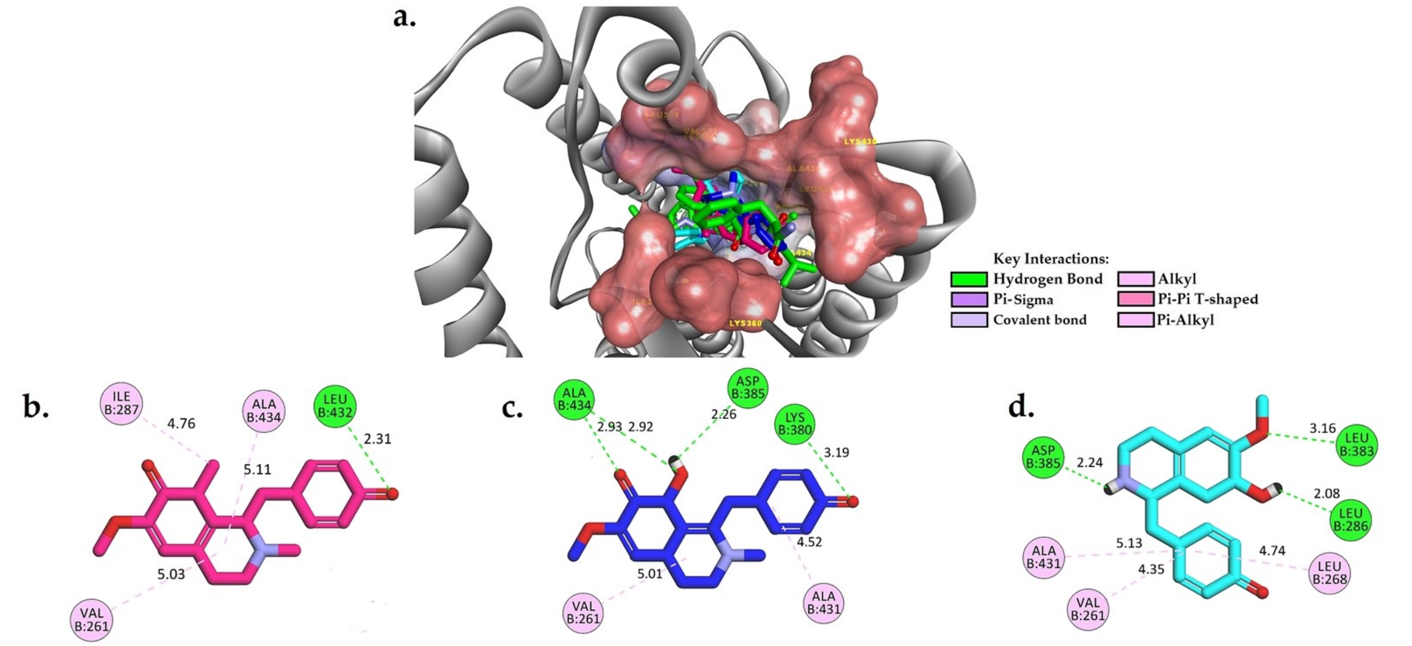

Figure S7. 3D binding poses interactions between L-685458 (green C, red O, pale blue N), cissamaline (pink C, red O, pale blue N), cissamanine (blue C, red O, pale blue N), and cissamdine (cyan C, red O, pale blue N) and gamma-secretase (GS) (a). 2D interaction analysis of cissamaline (b), cissamanine (c), and cissamdine (d) docked in the active binding site of GS, represented by a solid surface rendering with pKa color-coding. GS (PDB ID: 7D8X) is shown as a gray ribbon.
